# Risk of hip fractures in patients with depressive disorders: A nationwide, population-based, retrospective, cohort study

**DOI:** 10.1371/journal.pone.0194961

**Published:** 2018-04-11

**Authors:** Chih-Chuan Pan, Li-Yu Hu, Ti Lu, Ming-Shium Tu, Cheng-Che Shen, Zi-Jun Chen

**Affiliations:** 1 Center for Geriatrics and Gerontology, Kaohsiung Veterans General Hospital, Kaohsiung, Taiwan; 2 Department of Psychiatry, Kaohsiung Veterans General Hospital, Kaohsiung, Taiwan; 3 Division of Psychiatry, Faculty of Medicine, National Yang-Ming University, Taipei, Taiwan; 4 Department of Family Medicine, Pingtung Branch, Kaohsiung Veterans General Hospital, Pingtung, Taiwan; 5 Department of Psychiatry, Chiayi Branch, Taichung Veterans General Hospital, Chiayi, Taiwan; 6 Department of Information Management, National Chung-Cheng University, Chiayi, Taiwan; Garvan Institute of Medical Research, AUSTRALIA

## Abstract

**Background:**

Some studies have suggested that depressive disorders may play a vital role in the incidence of hip fractures. However, nationwide data are lacking regarding the association between depressive disorders and hip fractures.

**Objective:**

We aimed to explore the association between depressive disorders and new-onset hip fractures.

**Methods:**

We conducted a retrospective study of 11,207 patients with depressive disorders and 11,207 control patients using Taiwan’s National Health Insurance Research Database. A Cox regression model was used to evaluate the risk of hip fractures in patients with depressive disorders.

**Results:**

The incidence rate ratio of hip fractures between patients with depressive disorders and controls was 1.6 (95% confidence interval [CI] = 1.29–1.99, *P* < .001). After adjustment for potential confounders in multivariate analysis using the Cox regression model, patients with depressive disorders were found to have 1.34 times higher risk of hip fractures than controls (95% CI = 1.08–1.66, *P* = .008). Furthermore, age (hazard ratio [HR] = 7.43, 95% CI = 4.94–11.19, *P* < .001), hypertension (HR = 1.63, 95% CI = 1.17–2.28, *P* = .004), diabetes mellitus (HR = 1.47, 95% CI = 1.08–1.99, *P* = .014), cerebrovascular disease (HR = 1.76, 95% CI = 1.31–2.35, *P* < .001), living in rural areas (HR = 1.88, 95% CI = 1.30–2.70, *P* = .001), and low monthly income (NT$0–NT$19,000: HR = 4.08, 95% CI = 1.79–9.29, *P* = .001 and NT$19,100–NT$42,000: HR = 4.09, 95% CI = 1.76–9.49, *P* = .001) were independent risk factors for new-onset hip fractures in patients with depressive disorders.

**Conclusion:**

Depressive disorders might increase the risk of new-onset hip fractures, particularly in older patients and patients with hypertension, diabetes mellitus, cerebrovascular disease, or low socioeconomic status.

## Introduction

Depressive disorders are one of the most critical psychiatric illnesses affecting people in the 21^st^ century [1]. A study reported an increased risk of mortality in people with depressive disorders; therefore, depressive disorders should be regarded as a life-threatening disorder [2]. The etiology of depressive disorders is very complicated and considered to result from an interaction of multiple genes with environmental factors [3]. The neuronal circuit of serotonin, norepinephrine, and dopamine systems and their connections to specific areas in the brain are considered to be involved in depressive disorders [4, 5]; however, the exact underlying pathophysiological mechanism is unclear.

Hip fractures are a public health problem worldwide. Approximately 1.5 million hip fractures occur annually, and 3.9 million hip fractures are estimated to occur worldwide in 2050 [6]. A greater than 10-fold variation in hip fracture risk exists among countries [7]. Median age-standardized rates of hip fractures are the highest in North America and Europe; however, hip fracture rates are increasing in parts of Asia and Latin America [8]. The risk factors for hip fractures include age, sensory impairment, foot deformities, increased use of chronic medication, alcohol consumption, benzodiazepine use, environmental hazards at home, depression, muscular weakness, orthostatic hypotension, and impaired cognition [9].

Evidence supports an association between depressive disorders and the increased risk of fractures [10]. All types of falls are more likely to occur among patients with depressive disorders, thereby resulting in more hip fractures among these patients [11]. The previous study reported that depressive disorders might induce bone loss and osteoporotic fractures, primarily through specific immune and endocrine mechanisms, with poor lifestyle habits as potential contributory factors [12]. Moreover, numerous studies have confirmed multiple neuropathological changes in the brain of patients with depressive disorders. The neuropathological changes can influence the balance and gait coordination, which are regarded as important risk factors for fall-down accidents and hip fractures. Therefore, we hypothesized that a history of depressive disorders increases the risk of hip fractures.

Studies have revealed a positive correlation between depressive disorders and hip fractures [11, 13]. However, the participants enrolled were elderly, which may have limited the clinical impact and its generalizability. In addition, nationwide data are lacking, and most importantly, few longitudinal studies have reported the association between depressive disorders and new-onset hip fractures. A longitudinal study design would provide more information for clarifying the association between the depressive disorders and new-onset hip fracture. For example, a patient with a newly-diagnosed depressive disorder may have a higher risk because of unbearable psychic pain and associated depressive symptoms such as psychomotor retardation, difficulty in concentration or even inappropriate coping strategy by drinking alcohol. Furthermore, patients with first-episode depression may also have a greater possibility of receiving psychotropic sedative agents which they had never taken before. If the above-mentioned situations in newly-diagnosed depressed patients are the frequently-seen scenarios, then the timing of new-onset hip fracture would more likely be the first few days or months. However, if a new-onset hip fracture developed later than expected after diagnosis of a depressive disorder, then hypotheses such as immune and endocrine mechanisms or depression-related bone loss would be more reasonable explanations for the association between depressive disorder and new-onset hip fracture. Therefore, we conducted this population-based retrospective cohort study to investigate the possible association between depressive disorders and risk of hip fractures. Most importantly, we attempted to provide likely reasons to explain the possible association. Moreover, we aimed to determine independent risk factors for hip fractures among patients with depressive disorders.

## Materials and methods

### Data source

The National Health Insurance Research Database (NHIRD) in Taiwan, established in 1996, includes registry data and all medical benefit claims for approximately 99% of the citizens in Taiwan. The NHIRD contains abundant information regarding nearly all kinds of medical services, such as clinical outpatient visits (including the dates of visits), prescription medicine, and medical illnesses coded according to the International Classification of Diseases, Ninth Revision, Clinical Modification (ICD-9-CM) coding system. The dataset that we used in the study, called the Longitudinal Health Insurance Database 2000 (LHID 2000) was a part of the NHIRD. The LHID 2000 contains claims data of 1,000,000 beneficiaries who were randomly sampled from the NHIRD during January 1, 2000 to January 1, 2001. In addition, although the LHID 2000 was extracted from the NHIRD in the year 2000, the dataset of 1,000,000 beneficiaries included medical information from 1996 to 2013.

### Ethics statement

This study was evaluated and approved by the Institutional Review Board of Kaohsiung Veterans General Hospital. Written consent was not obtained because all the data in the LHID 2000 was de-identified.

### Study population

A retrospective cohort study was conducted using the data extracted from the LHID 2000. Patients who were newly diagnosed with depressive disorders between January 1, 2000 and December 31, 2004 were enrolled. Patients with depressive disorders were defined according to ICD-9-CM codes 296.2–296.3, 300.4, and 311. To ensure the accuracy of depressive disorder diagnosis, only subjects who were diagnosed with depressive disorders by a psychiatrist were selected. In both depressive disorder and control groups, we excluded patients who experienced hip fractures (ICD-9-CM code: 820.0, 820.2, and 820.8) before the enrollment date. Finally, we identified 11,207 patients with depressive disorders who met the eligibility criteria of the study. To develop a comparison cohort, we randomly selected 11,207 patients without a history of depressive disorders. These controls were matched with the study cohort at a ratio of 1:1 by age and sex, and using the same exclusion criteria over the same period. We defined the first date of depressive disorder diagnosis in the database as the index date and commenced follow-up of both patients with depressive disorders and controls. The primary clinical outcome was a new-onset hip fracture. All study patients and controls were observed until a new-onset hip fracture, death, withdrawal from the NHI system, or December 31, 2013.

### Statistical analysis

The incidence of new-onset hip fractures in the depressive disorder and control patients was calculated and stratified by sex and age (younger than 50 years or 50 years and older). Independent *t* tests and chi-squared test were used to examine the differences in the demographic characteristics of the depressive disorder and control patients. To investigate potential surveillance bias, patients were stratified into subgroups according to the duration since depressive disorder diagnosis.

Firstly, the Cox proportional hazard regression model was used to identify possible confounders and to exclude their effects on the process of evaluating whether depressive disorders increased the risk of new-onset hip fractures. The variables used in the univariate COX proportional hazard regression model were age, sex, degree of urbanization, monthly income, and common comorbidities. We explored the variables one by one and only the variables with *P* values < 0.1 in the univariate model were entered into the multivariable analysis. Secondly, we identified the variables that were potential predictors of hip fractures in patients with depressive disorders and therefore we repeated the univariate and multivariate Cox proportional hazard regression model again for the depression group only. The time frame used to obtain information for the comorbidities was from January 1, 1996 to the index date. The method that we used to define the comorbidities was also based on the ICD-9-CM system (hypertension: 401–405; coronary artery disease: 410–414; diabetes mellitus: 250; dyslipidemia: 272; cerebrovascular disease: 430–438; chronic obstructive pulmonary disease: 491, 492, 496; nephropathy: 580–589; chronic liver disease: 070.2, 070.3, 070.41, 070.44, 070.51, 070.54, 070.9, V02.61, V02.62, 571; autoimmune disease: 136.1, 340, 443.1, 446.0–446.2, 446.4–446.7, 555, 556, 694.4, 710.0–710.4, 714.0; osteoporosis: 733.0).

Statistical Analysis System for Windows, Version 9.3 (SAS Institute, Cary, NC, USA) was used for data analyses. All other statistical analyses were performed using Statistical Package for the Social Sciences for Windows, Version 20 (IBM, Armonk, NY, USA), except for the 95% CI was obtained by statistical software called MedCalc^®^.

## Results

### Patient selection

Our study sample comprised 11,207 patients with depressive disorders and 11,207 controls; the basic characteristics of both cohorts are presented in Table 1. The median age of both cohorts was 38.6 years (interquartile range, 28.6–50.9 years). The depressive disorder cohort showed a higher percentage of comorbidities and had a significantly lower income and a lower degree of urbanization than the control cohort.

**Table 1:** Baseline characteristics of patients with and without depressive disorders.

Demographic data		Patients with Depressive Disorders *n* = 11,207		Patients without Depressive Disorders *n* = 11,207		*P* value
		*n*	%	*n*	%	
Age (years)^a^		38.6 (28.6–50.9)		38.6 (28.6–50.9)		
	≥50	2,970	26.5	2,970	26.5	.999
	<50	8,237	73.5	8,237	73.5	
Sex, No. (%)						
	Male	4,504	40.2	4,504	40.2	.999
	Female	6,703	59.8	6,703	59.8	
Comorbidities, No. (%)						
	Hypertension	2,506	22.4	1,791	16.0	< .001
	Coronary artery disease	1,706	15.2	1,052	9.4	< .001
	Diabetes mellitus	1,596	14.2	1,080	9.6	< .001
	Dyslipidemia	2,072	18.5	1,353	12.1	< .001
	Cerebrovascular disease	1,293	11.5	722	6.4	< .001
	COPD	1,463	13.1	907	8.1	< .001
	Nephropathy	1,437	12.8	936	8.4	< .001
	Chronic liver disease	3,845	34.3	2,482	22.1	< .001
	Autoimmune disease	701	6.3	478	4.3	< .001
	Osteoporosis	735	6.6	446	4.0	< .001
Urbanization, No. (%)						< .001
	Urban	7,099	62.5	6,962	62.1	
	Suburban	3,220	28.7	3,559	31.8	
	Rural	888	7.9	686	6.1	
Monthly Income, No. (%)						< .001
	>NT$42,000^b^	1,272	11.4	1,344	11.9	
	NT$19,100–NT$42,000^b^	1,963	17.5	2,330	20.8	
	NT$0–NT$19,000^b^	5,506	49.1	5,175	46.2	
	Dependent	2,466	22.0	2,376	21.2	
Follow-up years^a^		4.92 (1.89–8.31)		7.32 (3.47–9.57)		< .001

^a^ Median age (interquartile range)

^b^ NT$1 = US$0.033.

COPD, chronic obstructive pulmonary disease.

### Incidence rate of hip fractures

As shown in Table 2, 227 patients with depressive disorders and 145 controls were diagnosed with hip fractures during the entire follow-up period. The incidence rate ratio (IRR) of hip fractures was significantly higher in the depressive disorder cohort than in the control cohort (IRR = 1.60, 95% confidence interval [CI] = 1.29–1.99, *P* < .001). Age stratification revealed a significantly higher IRR of hip fractures only among patients aged ≥50 years (IRR = 1.71, 95% CI = 1.35–2.17, *P* < .001). In addition, although the IRR of new-onset hip fractures remained significantly high in both women and men, stratification by follow-up durations revealed no significant differences in the incidence of hip fractures in any group (0–1, 1–5, 5–10, and ≥10 years).

**Table 2:** Incidence of hip fracture in patients with and without depressive disorders.

		Patients with Depressive Disorders		Patients without Depressive Disorders		Rate ratio (95% CI)	*P* value
		No. of Hip Fracture	Per 1,000 person-years	No. of Hip Fracture	Per 1,000 person-years		
Total		227	1.94	145	1.21	1.60 (1.29–1.99)	< .001
Age							
	≥50	189	6.89	118	4.04	1.71 (1.35–2.17)	< .001
	<50	38	0.42	27	0.30	1.42 (0.85–2.42)	.160
Sex							
	Male	92	2.03	58	1.23	1.65 (1.18–2.33)	.003
	Female	135	1.88	87	1.20	1.57 (1.19–2.08)	< .001
Follow-up, y							
	0–1	32	322.19	13	175.59	1.83 (0.94–3.81)	.061
	1–5	91	56.78	49	44.87	1.27 (0.88–1.83)	.183
	5–10	73	2.73	64	2.41	1.13 (0.80–1.61)	.460
	≥10	31	0.35	19	0.21	1.69 (0.93–3.17)	.067

CI, confidence interval

### Effects of depressive disorders on the risk of hip fractures

Table 3 shows the comparison of crude hazard ratio (HR) and adjusted HR (aHR) of new-onset hip fractures between patients with depressive disorders and controls. After adjustment for age, sex, comorbidities, degree of urbanization, and monthly income, multivariate analysis indicated a significantly higher risk of new-onset hip fractures in patients with depressive disorders (aHR = 1.34, 95% CI = 1.08–1.66, *P* = .008).

**Table 3:** Analyses of risk dactors for hip fracture in patients with and without depressive disorders.

Predictive variables		Univariate analysis		Multivariate analysis	
		HR (95% CI)	*P* value	HR (95% CI)	*P* value
Depressive disorders		1.60 (1.30–1.97)	< .001	1.34 (1.08–1.66)	.008
Age (≥50 = 1, <50 = 0)		15.2 (11.6–19.8)	< .001	6.89 (5.04–9.41)	< .001
Sex (Female = 1, Male = 0)		0.95 (0.77–1.16)	.590	0.99 (0.79–1.24)	.936
Comorbidities					
	Hypertension	8.20 (6.65–10.12)	< .001	1.89 (1.46–2.45)	< .001
	Coronary artery disease	5.24 (4.23–6.43)	< .001	1.11 (0.87–1.42)	.396
	Diabetes mellitus	4.72 (3.82–5.84)	< .001	1.37 (1.08–1.76)	.011
	Dyslipidemia	3.48 (2.82–4.30)	< .001	0.85 (0.66–1.09)	.197
	Cerebrovascular disease	6.47 (5.22–8.01)	< .001	1.75 (1.38–2.22)	< .001
	COPD	3.98 (3.18–4.99)	< .001	1.34 (1.05–1.70)	.019
	Nephropathy	3.04 (2.40–3.85)	< .001	1.20 (0.93–1.55)	.152
	Chronic liver disease	1.92 (1.56–2.36)	< .001	0.94 (0.75–1.18)	.598
	Autoimmune disease	2.12 (1.51–2.98)	< .001	1.38 (0.98–1.95)	.064
	Osteoporosis	4.57 (3.53–5.91)	< .001	1.29 (0.97–1.70)	.079
Degree of urbanization				
	Urban	Reference			
	Suburban	1.07 (0.92–1.24)	.002	1.16 (0.92–1.46)	.203
	Rural	1.15 (0.92–1.42)	< .001	1.63 (1.21–2.21)	.001
Monthly Income				
	>NT$42,000^a^	Reference		Reference	
	NT$19,100–NT$42,000^a^	7.41 (3.76–14.60)	< .001	4.01 (2.02–7.96)	< .001
	NT$0–NT$19,000^a^	6.74 (3.46–13.12)	< .001	4.34 (2.22–8.50)	< .001
	Dependent	1.02 (0.45–2.32)	.969	1.16 (0.51–2.66)	.722

^a^ NT$1 = US$0.033.

HR, hazard ratio; CI, confidence interval; COPD, chronic obstructive pulmonary disease.

### Risk factors for hip fractures in patients with depressive disorders

Table 4 demonstrates that age (HR = 7.43, 95% CI = 4.94–11.19, *P* < .001), hypertension (HR = 1.63, 95% CI = 1.17–2.28, *P* = .004), diabetes mellitus (HR = 1.47, 95% CI = 1.08–1.99, *P* = .014), cerebrovascular disease (HR = 1.76, 95% CI = 1.31–2.35, *P* < .001), living in rural areas (HR = 1.88, 95% CI = 1.30–2.70, *P* = .001), and low monthly income (NT$0–NT$19,000: HR = 4.08, 95% CI = 1.79–9.29, *P* = .001 and NT$19,100–NT$42,000: HR = 4.09, 95% CI = 1.76–9.49, *P* = .001) were independent risk factors for new-onset hip fractures in patients with depressive disorders.

**Table 4:** Analyses of risk factors for hip fracture in patients with depressive disorders.

Predictive variables		Univariate analysis		Multivariate analysis	
		HR (95% CI)	*P* value	HR (95% CI)	*P* value
Age (≥50 = 1, <50 = 0)		16.32 (11.52–23.13)	< .001	7.43 (4.94–11.19)	< .001
Sex (Female = 1, Male = 0)		0.93 (0.71–1.21)	.573	0.98 (0.74–1.31)	.890
Comorbidities					
	Hypertension	7.34 (5.60–9.64)	< .001	1.63 (1.17–2.28)	.004
	Coronary artery disease	4.66 (3.58–6.07)	< .001	1.04 (0.77–1.40)	.816
	Diabetes mellitus	4.71 (3.62–6.14)	< .001	1.47 (1.08–1.99)	.014
	Dyslipidemia	3.46 (2.66–4.51)	< .001	0.93 (0.68–1.27)	.660
	Cerebrovascular disease	6.09 (4.66–7.94)	< .001	1.76 (1.31–2.35)	< .001
	COPD	3.43 (2.59–4.55)	< .001	1.26 (0.94–1.71)	.128
	Nephropathy	2.76 (2.06–3.70)	< .001	1.17 (0.85–1.60)	.339
	Chronic liver disease	1.80 (1.39–2.34)	< .001	0.95 (0.72–1.26)	.711
	Autoimmune disease	1.81 (1.17–2.78)	.007	1.20 (0.77–1.85)	.424
	Osteoporosis	4.45 (3.26–6.08)	< .001	1.32 (0.94–1.86)	.109
Degree of urbanization				
	Urban	Reference		Reference	
	Suburban	1.46 (1.09–1.97)	.012	1.20 (0.89–1.62)	.243
	Rural	3.41 (2.40–4.83)	< .001	1.88 (1.30–2.70)	.001
Monthly Income				
	>NT$42,000^a^	Reference		Reference	
	NT$19,100–NT$42,000^a^	6.88 (2.99–15.80)	< .001	4.09 (1.76–9.49)	.001
	NT$0–NT$19,000^a^	5.82 (2.57–13.18)	< .001	4.08 (1.79–9.29)	.001
	Dependent	0.77 (0.26–2.28)	.634	0.93 (0.31–2.77)	.895

^a^ NT$1 = US$0.033.

HR, hazard ratio; CI, confidence interval; COPD, chronic obstructive pulmonary disease

## Discussion

The major finding of our study using a nationwide population-based data set was that depressive disorders were significantly associated with an increased risk of new-onset hip fractures. In addition, older age (≥50 years), hypertension, diabetes mellitus, cerebrovascular disease, and lower socioeconomic status may be risk factors for new-onset hip fractures in patients with depressive disorders.

Studies have provided evidence for a positive correlation between depressive disorders and hip fractures [10, 11, 14, 15]. Hwang et al. (2011) reported that depressive disorders were associated with a 2.85-fold increased risk of hip fractures [13]. These findings are consistent with the present findings. However, our study benefited from a nationwide population-based longitudinal analysis, with a large sample size and longer follow-up duration. Furthermore, participation in the NHI program is mandatory, and all Taiwanese residents can access low-cost health care, referral bias is small, and follow-up compliance of patients is high.

The exact mechanism underlying depressive disorders and new-onset hip fractures is unclear. Several possible pathophysiological factors may explain this association. First, low bone mineral density in depressive disorders may result in new-onset hip fractures. Numerous studies have provided evidence that depressive disorders adversely affect bone density and increase the risk of fracture [12, 16, 17]. A study suggested that a high level of parathyroid hormone plays a role in the pathophysiological process of osteopenia in patients with depressive disorders [18]. Other possible mechanisms that might elucidate the association between low bone mineral density and depressive disorders include involvement of the hypothalamic–pituitary–adrenocortical and the sympathoadrenal axes, cytokines, immune responses, alcohol consumption, poor nutritional status, social circumstances, and an unhealthy lifestyle [16, 19–21]. In addition, depression tends to develope sedentary lifestyle and decrease outdoor activity [22], which may cause less sun exposure. As we know, less sun exposure is associated with vitamin D deficiency and then increases the risk of osteoporosis [23]. In our analysis, osteoporosis was not a significant confounder for hip fractures in both cohorts, the reason for the different finding needs further exploration. Second, antidepressants, which are widely used by patients with depressive disorders, may play a crucial role in hip fractures [24–26], particularly in geriatric patients. This observation is consistent with our finding that age (≥50 years) is a significant risk factor for hip fractures in patients with depressive disorders. Bakken et al. (2013) reported an increased risk of hip fractures in people exposed to antidepressants, particularly those with serotonergic properties such as selective serotonin reuptake inhibitors [27]. Studies have suggested that drugs of all classes (first- and second-generation antidepressants) are associated with a higher risk of hip fractures [28, 29]. Third, neuropathological changes in certain regions of brain in patients with depressive disorders may be associated with higher risk of falls and hip fractures. Depressive disorders are characterized by structural and functional abnormalities in the frontal lobe, basal ganglia, hippocampus, and amygdala [30]. In addition, studies have reported a reduction in the gray matter volume of the putamen [31], excess neuronal loss [32], morphological atrophy, vascular atherosclerosis, and neurodegenerative changes [33]. These pathophysiological problems of the brain would influence the balance, judgment, and gait coordination of patients with depressive disorders, and are considered to be related to a higher risk of falls and hip fractures.

Another finding of this study was that older age, hypertension, diabetes mellitus, cerebrovascular disease, living in rural areas, and low monthly income are risk factors for hip fractures in patients with depressive disorders. As mentioned previously, age is a risk factor for hip fractures [9], which may explain the present findings. The effects of antihypertensive drugs on hip fractures may account for the increased risk of hip fractures in patients with hypertension. The risk of hip fractures increased during the initiation period of antihypertensive drugs, probably because of orthostatic hypotension-related falls [34, 35]. Studies have reported changes in bone metabolism and decreased bone mineral density following long-term treatments with some antihypertensive drugs [35–37], causing more hip fractures. Furthermore, studies have suggested possible mechanisms for the higher fracture risk in patients with diabetes mellitus. The complications of diabetes mellitus, including hypoglycemia, diabetic retinopathy, and peripheral neuropathy, may increase the risk of falling [38–40]. Studies have reported deterioration in bone microarchitecture and quality in patients with diabetes, which lead to increased fractures [38, 41]. Furthermore, studies have demonstrated an increased risk of hip fractures after stroke, probably because of an increased fall risk and decreased femoral bone mineral density after stroke [42–44], which may explain why cerebrovascular disease is a risk factor for new-onset hip fractures. In this study, we observed a higher risk of hip fractures in patients with depressive disorders who live in rural areas and have a lower income. The possible reasons for this finding may be complicated. Patients with depressive disorders who live in rural areas and have lower income might have poor nutritional status, inadequate dietary calcium intake, lower vitamin D levels, lower bone mineral density, higher osteoporosis prevalence, and less opportunity to receive adequate treatment [45–50]; all these factors may result in increased hip fractures. Previous evidence supports osteoporosis is a risk factor for hip fractures in patient with depressive disorders, but the result was not shown in our analysis. The possible cause is the use of ICD-9-CM code to define osteoporosis in our analysis. There is the possibility of underestimation especially in the young age and male gender because of the health policy and only patients seeking medical services could be identified.

Our study is one of the few nationwide cohort studies to examine depressive disorders as a risk factor for hip fractures. However, several limitations inherent to the use of claims databases should be considered. First, several essential information such as tobacco or alcohol use, lifestyle, quality of living environment, and family support are lacking in the NHIRD. Analysis of these variables may provide useful information regarding additional risk factors linking depressive disorders and hip fractures. Second, the severity of depressive disorders and the pharmaceutical data including other psychosocial treatment strategies for depressive disorders were not analyzed in our study, and this might be related to the risk of new-onset hip fractures. Third, the fracture type such as the major fracture or fragility fracture are lacking. The mechanism and risk factors for major fracture or fragility fracture are different, but we could not analyze due to the limitation. Fourth, data from the NHIRD were used for government health service billing and were not verified by scientific studies. Finally, we used ICD-9-CM codes to define depressive disorders and hip fractures; the prevalence may be underestimated because only patients seeking medical services could be identified using the NHIRD, which may result in the underestimation of the association of depressive disorders with hip fractures.

## Conclusion

Patients with depressive disorders have a significantly higher risk of hip fractures after adjustment for selective confounders such as age, sex, and underlying medical comorbidities. Furthermore, the rate ratios are higher in patients who are older (≥50 years), have either hypertension, diabetes mellitus, or cerebrovascular disease, live in rural areas, and have lower income. Adequate prevention strategies are essential to reduce the incidence of hip fractures in these high-risk patients with depressive disorders. Further prospective clinical studies on the relationship between depressive disorders and hip fractures are warranted.
